# Circular Potential of Lithium‐Ion Battery Recycling Slags: Quantifying Microstructure and Elemental Distribution for a Holistic Valorization

**DOI:** 10.1002/advs.202523988

**Published:** 2026-02-11

**Authors:** Peter Cornelius Gantz, Charlize Alexia Senkyr, Rüdiger Kilian, Andreas Neumann, Maximilian Korges, Daniel A. Frick, Hans Roggendorf, Ralf Wehrspohn, Stefan Stöber, Christiane Stephan‐Scherb

**Affiliations:** ^1^ Institute for Geosciences and Geography Martin‐Luther‐University Halle‐Wittenberg Halle (Saale) Germany; ^2^ ITEL – Institute for Technologies and Economics of Lithium GmbH Halle (Saale) Germany; ^3^ Institute for Geosciences University of Potsdam Potsdam‐Golm Germany; ^4^ Institute for Geosciences Kiel University Kiel Germany; ^5^ Institute for Physics Martin‐Luther‐University Halle‐Wittenberg Halle (Saale) Germany

**Keywords:** β‐eucryptite, circularity, EnAM, in‐situ chemical characterization, metal recovery, quantitative microstructural analysis

## Abstract

Extractive pyrometallurgy is an established process for recycling lithium‐ion batteries (LIB). One approach to recover lithium from slags produced by pyrometallurgical recycling of LIB involves accumulating lithium in an engineered artificial mineral (EnAM) facilitating recovery. β‐Eucryptite (LiAlSiO_4_) is a promising EnAM‐candidate as it is chemically similar to the primary lithium ore spodumene (LiAlSi_2_O_6_) making co‐processing a viable option. However, for economic recycling the β‐eucryptite must have a high lithium content, a high purity, and display a favorable microstructure. The presented work aims to quantify all three parameters. An industrial pyrometallurgical slag was analyzed using scanning electron microscopy (SEM), electron probe microanalysis (EPMA) and laser ablation inductively coupled plasma mass spectroscopy (LA‐ICP‐MS). The analyzed β‐eucryptite shows a high lithium content of 5.45 mass % while containing only low amounts of iron (0.64 mass %) and calcium (0.2 mass %). However, small mean grain sizes (< 21 µm) and unfavorable grain shapes impede the separation of β‐eucryptite. It was discovered that chromium and vanadium were accumulated in spinel phases of the chromite‐coulsonite solid solution series. Producing a Cr, V‐bearing spinel concentrate would enable the recycling of both metals as well as improving the quality of the residual lithium‐depleted slag as a by‐product.

## Graphical abstract

A lithium‐bearing slag is investigated with the goal of holistic valorization. The present β‐eucryptite (LiAlSiO_4_) exhibits a high lithium content and low levels of impurities. The spinel contains most of the chromium and vanadium, representing additional valorization opportunities. However, both phases show low grain sizes and an unfavorable microstructure, emphasizing the importance of microstructural optimization of EnAM‐phases.

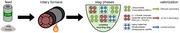

## 1 Introduction

In the context of the ongoing electrification of the mobility sector, lithium‐ion batteries (LIB) experience an increasing demand [1]. LIBs are currently the battery technology with the highest energy density and are steadily engineered to reach even higher capacities [2]. The EU battery directive [3] and the demand for resource efficiency require fast and efficient solutions for secondary metal recovery. Extractive pyrometallurgy solves nickel, cobalt, and copper recovery from spent LIB; however, lithium dissolves in the slag from where it can currently not be recovered economically, an issue that needs to be addressed [4, 5]. An approach to recover lithium from the slag, recently discussed by the scientific community, is the “Engineering of Artificial Minerals” (EnAM) strategy [6, 7, 8, 9]. This strategy employs lithium collector phases with a high lithium content, which form during the cooling of the slag. β‐Eucryptite was established as a promising and easy to implement EnAM‐phase in our previous work [10]. Interestingly, due to its high lithium‐ion conductivity, β‐eucryptite could also appear in the upstream of the battery lifecycle as it is currently also investigated as a potential electrolyte for solid‐state LIB, which will enter the market in the foreseeable future [11, 12].

Combining primary and secondary resource streams represents a potential efficient solution for lithium recycling as this is already standard for other important metals [13]. The goal of this paper is to analyze the prerequisites of co‐processing secondary β‐eucryptite with primary spodumene concentrate, an approach with vast potential for economical lithium recovery by enabling the benefits of economy of scale as spodumene is the most commonly used lithium ore mineral [14]. For the lithium refining from spodumene, a concentrate, produced by flotation of spodumene‐bearing, crushed and milled pegmatite rock, is roasted to around 1050 °C to convert α‐spodumene to the more chemically reactive β‐spodumene [15]. The β‐spodumene is leached with concentrated sulfuric acid, producing Li_2_SO_4_ and leach residue [15, 16].

Chemically, β‐eucryptite (LiAlSiO_4_) has a related composition to spodumene (LiAlSi_2_O_6_), making it a premiere candidate for co‐processing. Additionally, β‐eucryptite is leachable without roasting. Hence, a β‐eucryptite concentrate can likely be applied right before the leaching step, which is cost efficient as the thermal treatment step can be skipped [17, 18]. Therefore, β‐eucryptite has potential benefits compared to other discussed lithium‐EnAM like γ‐lithiumaluminate (LiAlO_2_) [6, 7, 8, 9]. This advantage could be necessary to consider it a viable option, as the lithium content of β‐eucryptite is significantly lower than in other discussed lithium‐EnAM [8, 10].

As a basic condition for the recovery of lithium using a lithium‐EnAM, enough of the EnAM‐phase must form during the cooling of the slag. A β‐eucryptite concentrate must be produced from the slag to enable co‐processing with spodumene. To achieve a well‐sorted and lithium‐rich β‐eucryptite concentrate, specific criteria must be met:

1. High lithium content of the β‐eucryptite, close to its stoichiometric maximum,
2. A microstructure suitable for efficient liberation of β‐eucryptite, characterized by large, convex crystals and little intergrowth,
3. High purity, referring to little foreign ions incorporated in the crystal structure of β‐eucryptite.

**FIGURE 1:**
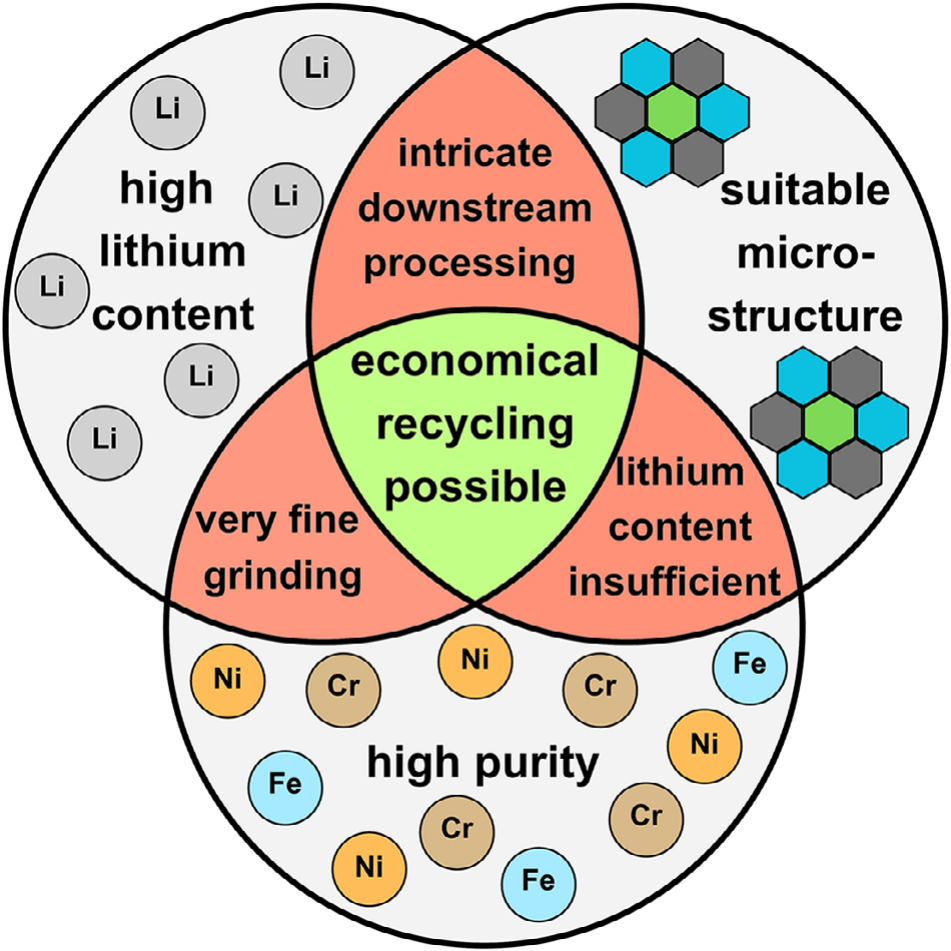
Influence of the lithium content, microstructure and impurities on the economical recyclability of a lithium‐EnAM. “Suitable microstructure” refers to large crystal sizes, fully convex crystal shapes, and low degree of intergrowth while impurity refers to the incorporation of foreign ions into the crystal structure.

These parameters are crucial for a recycling process based on any EnAM concentrate, i.e. even if one of these prerequisites is insufficient, a cost‐efficient lithium recovery becomes challenging. For example, if the lithium‐EnAM has an advantageous microstructure and a high lithium content, but contains elevated amounts of impurities, the multi‐step hydrometallurgy required to remove the impurities could impede an economical lithium recovery (Figure 1). Additionally, investigating the lithium distribution between the major slag phases is important to assess the enrichment of lithium in the lithium‐EnAM. A low lithium enrichment results in high lithium losses to the non‐lithium containing phases, resulting in an inefficient recovery process.

The focus of this study lies on investigating and quantifying these threshold parameters; therefore, the chemical composition of the slag minerals was determined by electron probe microanalysis (EPMA) and laser‐ablation inductively‐coupled‐plasma mass‐spectrometry (LA‐ICP‐MS). The microstructure of the slag, with a focus on the β‐eucryptite, was investigated using scanning electron microscopy (SEM) with a back‐scattered electron detector (BSE).

**FIGURE 2:**
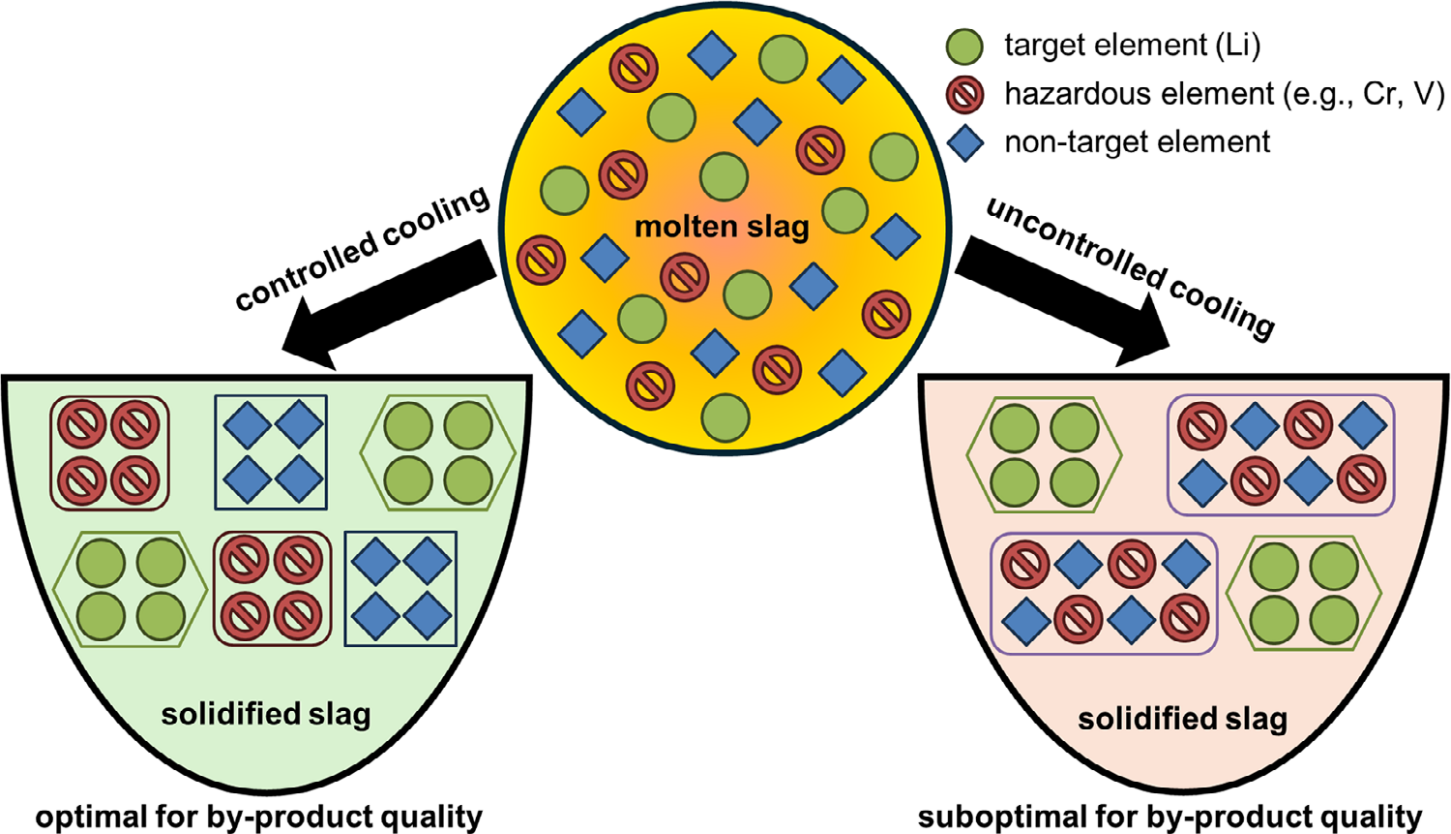
Schematic overview of the engineering of artificial minerals (EnAM) approach, including the enhancement of by‐product quality by accumulating hazardous elements in a target EnAM‐phase.

To achieve a process applicable in a circular economy, the by‐product, e.g. a ground lithium‐depleted slag, must be of sufficient quality to be used as a valuable resource in other industries, for example in construction. Hence, the secondary objective of the present study is to improve the quality of the by‐product. Applying the EnAM‐strategy in the context of undesired element removal is a currently missed opportunity. Therefore, this study will also focus on the distribution of potentially hazardous elements (e.g., chromium, vanadium) to explore options for their removal from the lithium‐depleted slag (Figure 2).

## 2 Experimental Section

### 2.1 Sample Preparation

**FIGURE 3:**
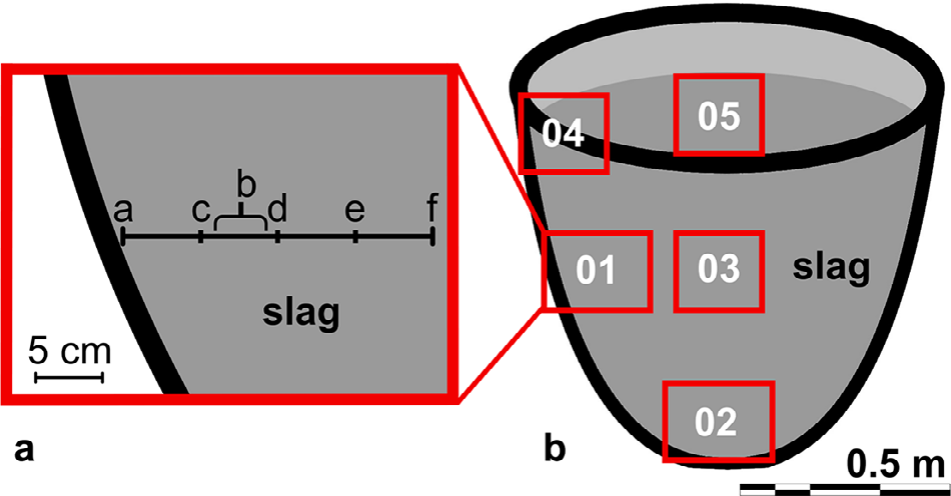
Sampling scheme of the slag cone (b) and subsampling positions at sample 01 (a). Subsample 01a represents the most outside part of sample 01 while subsample 01f was taken at a depth of 20 cm. Subsample 01b was taken between subsamples 01c and 01d.

A pyrometallurgical slag from the industrial LIB recycling process of Nickelhütte Aue GmbH was systematically sampled to gain insight into the chemical and mineralogical composition of the entire slag cone. The slag was air cooled under ambient conditions and has a complex chemical composition with SiO_2_, CaO, and Al_2_O_3_ as main components [10]. Further, the slag contains 3.40 mass % V_2_O_5_, 1.77 mass % Li_2_O, and 1.14 mass % Cr_2_O_3_ [10]. Additional information about the sampling process and the sample material were extensively described in Gantz et al. [10]. Additionally, thin sections were prepared, whereby representative subsamples were arbitrarily cut and glued on a glass sample holder using epoxy resin and polished to a thickness of 25 µm. For sample 01, five additional thin sections were produced. Subsamples were cut out every 5 cm in an arbitrary orientation, starting with 01a at 0 cm and reaching a depth of 20 cm with sample 01f (Figure 3). All thin sections were coated with carbon using the CCU‐010 (Safematic) to an approximate thickness of 10 nm.

### 2.2 SEM

A TESCAN Clara Scanning Electron Microscope (SEM) (Tescan Group, Brno, Czech Republic) equipped with a field emission gun (FEG) and energy dispersive X‐ray (EDX) detector was used for the backscattered electron (BSE) imaging. The imaging was performed at an accelerating voltage of 20 kV and a beam current of 800 pA. The dwelling time was set to 10 µs and a pixel size of 195 nm was achieved using a working distance of 15 mm. For subsequent image analysis, larger BSE‐mosaic images were produced by stitching the individual BSE‐images, producing total areas in the range of 13.2 and 22.9 mm^2^.

Additionally, EDX maps were taken with an accelerating voltage of 15 kV, a beam current of 800 pA, a dwelling time of 3.2 µs, with a pixel size of 120 nm, and at a working distance of 9.9 mm.

### 2.3 EPMA

The JEOL JXA‐8200 (Jeol Ltd., Akishima, Japan) at University of Potsdam was used for the electron probe microanalysis (EPMA). It was equipped with 5 wavelength dispersive spectrometers (WDX) set up with separate analyzer crystals for quantitative in situ chemical analysis. Light elements like lithium could not be measured using WDX. All phases were measured with an excitation voltage of 15 kV. To achieve good statistics, no more than one measuring spot was set at each grain.

The calibration was performed with external standards. The standards used to calibrate the individual elements were shown in the supporting information in Table S2. The calibration was verified at the start and at the end of each measuring program.

### 2.4 LA‐ICP‐MS

Laser‐ablation inductively‐coupled‐plasma mass spectrometry (LA‐ICP‐MS) for in situ quantitative lithium analysis of the individual slag minerals were conducted at the Institute of Geosciences at Kiel University (Kiel, Germany) using a Coherent GeoLas HD ArF 193 nm excimer laser system (Coherent, Saxonburg, PA, USA) coupled to an Agilent 8900 ICP‐MS/MS (Agilent Technologies, Santa Clara, CA, USA) operated in single quad mode. The samples were placed in a low dispersion high capacity laser ablation cell (LDHCLAC, ETH Zürich, see Fricker et al) [19].

**FIGURE 4:**
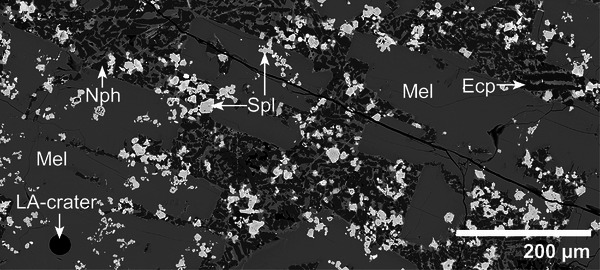
Exemplary BSE‐image of a thin section from sample 03 with a LA‐crater. Typical microstructure of the slag with melilite (Mel), spinel (Spl), nepheline (Nph), and β‐eucryptite (Ecp).

The instrument was tuned for high sensitivity, while keeping the oxide formation rate low (typically ThO/Th ≤ 0.4%) and the plasma robust (U/Th ≈ 1, on NIST SRM612). The measurements were performed using a fluence of the laser of around 8 J cm^−2^, a pulse frequency 10 Hz for a total of 400 pulses. Helium was used as carrier gas at a flow rate of 1011 mL min−1. The crater size was set to 24 µm (Figure 4) to ensure the highest possible spatial accuracy while enabling the measurement of very small grains, commonly present in the samples from the rim of the slag cone. The calibration was based on NIST SRM612 glass standard according to Jochum et al. [20] and OREAS999 prepared in‐house as nano pellet according to Garbe‐Schönberg and Müller [21] using 100wt oxide normalization approach [22], data evaluation was performed with the software LADR [23]. Additional secondary reference materials (USGS BCR‐2G, NIST SRM182 and SRM183) were analyzed throughout the session and the results compared to published values [24].

### 2.5 Microstructural Methods

The microstructure of the β‐eucryptites and the spinels was investigated with the goal to quantify the volume fraction and quantitatively describe their grain shape and grain size distribution. A detailed step‐by‐step documentation of the image analysis steps was provided in the supporting information (Table S1). The image analysis was carried out using the open‐source software Fiji was just ImageJ v1.54p [25].

The grain shape was quantitatively analyzed by implementing three shape descriptors. The aspect ratio (Equation 1) was chosen as a measure for the elongation of a grain [26, 27].

(1) $$Aspect\;ratio=\frac{a\;}{b}\;=\frac{length}{width}$$

The envelope parameters were selected to quantify lobateness and indentations of the grains by calculating *deltA* and *deltP* according to Equations (2) and (3) with *ΔP* = *P* – *PE*; *ΔA* = *AE* – *A*; *P* = perimeter of the grain, *PE* = perimeter of the convex hull; *A* = area of the grain, *AE* = area envelope of the convex hull. *DeltP* gives a percentage on how much of the perimeter of a grain can be attributed to its shape deviating from its convex hull (excess perimeter), while *deltA* gives a percentage of how much bigger the area of the convex hull was compared to the area of the grain (excess area). Thus, *deltP* and *deltA* provide more conclusive data to accurately depict grain shape than common measures like circularity or roundness [27, 28]. The calculation was done using the “Jazy_Env_map” macro [29]. Based on the way Fiji calculates the perimeter of particles, very small particles (< 27 px ≈ 1 µm^2^) tend to give extreme values. Hence, they were removed in preparation for the shape analysis only.

(2) $$deltP=\frac{\Delta P}{P}\;*100\%$$

(3) $$deltA=\frac{\Delta A}{A}\;*100\%$$

As a measure for grain size, the diameter of the area equivalent circle (eq. diameter) was used as the basis for calculating the 3D grain size distribution and to split up the data into two grain size fractions. Additionally, the Feret‐Min diameter (*f_min_*) was chosen as it was defined as the smallest measured diameter of a particle. Hence, it was comparable to a grain size practically produced by sieving, as sieving also selects according to the smallest diameter of a grain [30]. The area‐weighted modes of *f_min_* as well as the amount of phase with a *f_min_* of greater than 10 µm were calculated using a simple Python code.

**FIGURE 5:**
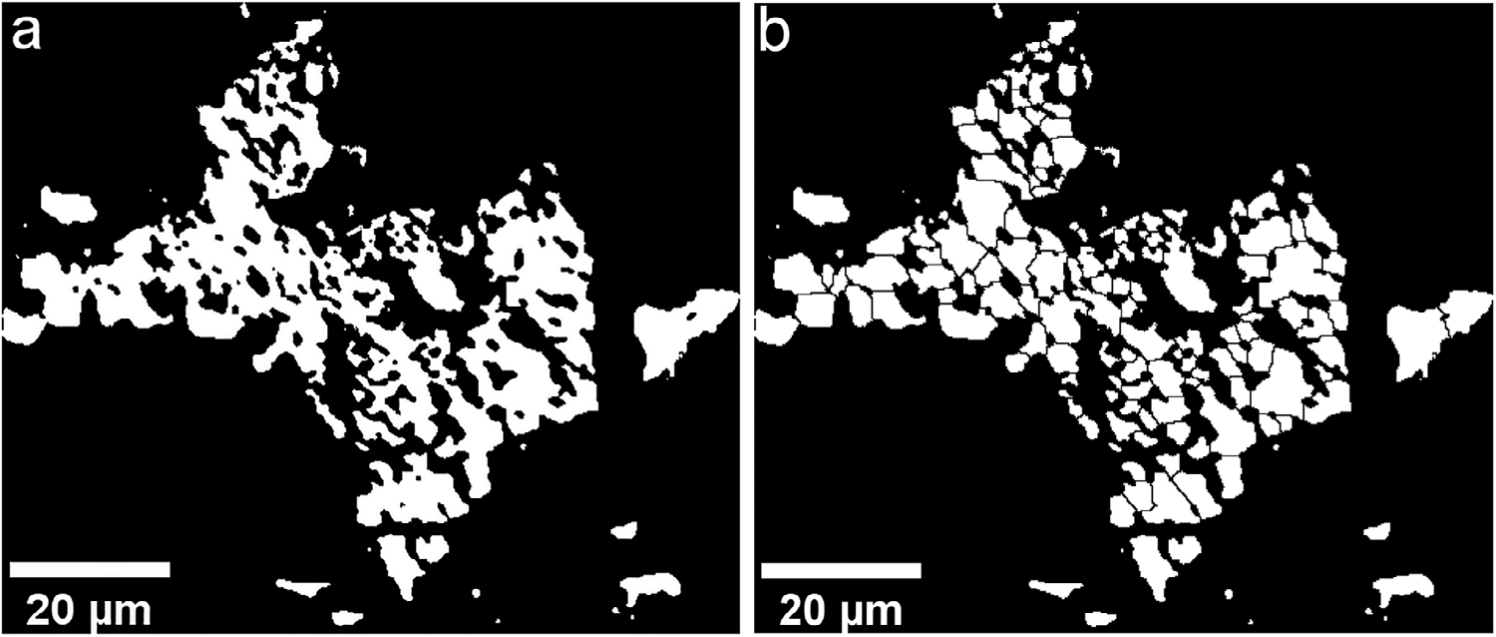
(a) Highly lobate β‐eucryptite grain. (b) Same grain after applying a watershed algorithm to estimate breakage of the grain during comminution.

The 3D grain size distribution (vD) was calculated using the iterative Schwartz‐Saltykow approach [29]. This method uses the 2D distribution of area sections, produced by cutting 3D grains, as input data. The calculation was done using the “Jazy_stripper” macro [29]. As the result of this calculation depends on the histograms representing the 2D distribution, the same bin width was chosen for all histograms. To estimate grain sizes after comminution the “Watershed Irregular Features” of the Fiji Plugin “BioVoxxel Toolbox” was used [31]. This function iteratively erodes a phase map, identifies if a new connection between background areas was created and dilates as much as it previously eroded, without reconnecting separated grains (Figure 5).

The error that comes with the quantification of volumes based on the area‐fraction was calculated for each phase individually and in accordance with Equations (4) & (5) with σ = standard deviation of particle areas, ∼a = average area of a particle, *n* = number of particles, ϕ = measured area fraction, *m* = an integer referring to the desired confidence level (e.g., 1, 2, 3), δ_*rel*_ = relative error, Δϕ_*m*σ_ = absolute error. The practical computation of the error was done using the “Jazy_Error” macro [29].

(4) $$\delta_{rel}=\frac{\sqrt{{\left(\frac{\sigma }{∼a}\right)}^{2}+1}}{\sqrt{n}}$$

(5) $$\Delta \;\phi_{m}=m*\phi *\delta_{rel}$$

## 3 Results and Discussion

### 3.1 Chemical Composition of the Slag Phases

The general chemical composition of the individual phases was determined by EPMA. However, the EPMA results of all phases with a significant lithium content, e.g. β‐eucryptite and lithium metasilicate, yielded a total of < 100 mass %. Deriving a semiquantitative lithium content using the mass balance gives only a first approximation, as other elements like hydrogen and fluorine were not quantified either. Therefore, the lithium content of all major phases, forming sufficiently sized crystals, was determined using LA‐ICP‐MS. For the β‐eucryptite the mass balance method of lithium determination is assessed against the LA‐ICP‐MS data.

#### 3.1.1 Composition of the Lithium‐Containing Phases

##### 3.1.1.1 Results of the EPMA measurements

The β‐eucryptite shows a very constant mineral chemistry with low standard deviations of each main component (Table 1). Furthermore, only minor amounts of foreign cations (e.g., Fe^2+^, Ca^2+^) are incorporated into the crystal structure. The total average impurity content, as the sum of all measured, non‐compositional oxides, of the β‐eucryptite is 1.26 mass % ± 0.77. However, it does show a slight non‐stoichiometric Al : Si ratio. Ideally, eucryptite has an atomic ratio of Al to Si of 1:1, corresponding to 40.45 mass % Al_2_O_3_ and 47,68 mass % SiO_2_ [32]. The measured β‐eucryptite shows the stoichiometric Al_2_O_3_ content but a slightly higher SiO_2_ content resulting in an atomic Al/Si ratio of 0.93 which is not uncommon for β‐eucryptite. However, this usually results in a lower lithium content, as Al is substituted together with Li in a coupled substitution (Equation (6) [32].

(6) $${\mathrm{Si}}^{4+}⇆\;{\mathrm{Al}}^{3+}+{\mathrm{Li}}^{+}$$

**TABLE 1:** Composition of the lithium‐bearing minerals measured by EPMA (mass %) with standard deviation in brackets and number of measured grains (n).

	β‐Eucryptite	Lithium metasilicate
n	74	5
SiO_2_	51.05 (0.92)	67.23 (0.65)
TiO_2_	0.01 (0.02)	0.03 (0.02)
Al_2_O_3_	40.47 (0.64)	1.76 (0.88)
Cr_2_O_3_	0.03 (0.03)	0.05 (0.01)
V_2_O_3_	0.06 (0.07)	0.49 (0.1)
FeO	0.64 (0.24)	2.36 (0.15)
MnO	0.09 (0.04)	3.1 (0.39)
MgO	0.06 (0.04)	0.69 (0.13)
CaO	0.2 (0.2)	0.77 (0.43)
ZnO	0.09 (0.06)	0.14 (0.12)
BaO	0.02 (0.03)	0.07 (0.06)
Na_2_O	0.05 (0.04)	0.31 (0.23)
K_2_O	< LOD	0.11 (0.05)
Cl	< LOD	0.03 (0.04)
Total	92.77 (0.91)	77.14 (1.22)

The lithium metasilicate incorporates a variety of foreign cations into its crystal structure. The total average impurity content is 9.91 mass % ± 2.61, which is nearly eight times higher compared to the β‐eucryptite. The measured SiO_2_ content (Table 1) is close to the stoichiometric lithium content of lithium metasilicate (Li_2_SiO_3_), containing 66.78 mass % SiO_2_; consequently, suggesting an incorporation on the lithium position, reducing the lithium content of the phase.

No lithium metasilicate crystals with diameters large enough for reliable LA‐ICP‐MS analysis, < 24 µm, could be found in the slag. Hence, the lithium content must be approximated using the mass balance of the EPMA analysis, resulting in a maximum lithium content of 10.6 mass %. Stoichiometric lithium metasilicate contains 15.3 mass % lithium, which is significantly higher. This discrepancy further supports the incorporation of foreign ions on the lithium position in the lithium metasilicate. The measured lithium metasilicate contains notable amounts of Al_2_O_3_, V_2_O_5_, FeO, MnO, MgO, CaO, ZnO, Na_2_O, and K_2_O (Table 1). There are two substitutions possible, leading to a lower overall lithium content:

1. Monovalent cations (e.g., Na^+^, K^+^) can be incorporated by a simple substitution of lithium (Equation (7),
2. To incorporate the divalent cations, two substitutions are possible: creating a vacancy (Equation (9) or as a coupled substitution with a trivalent cation replacing a lithium and a silicon (Equation 11).

Equations (8), (10), (12), and (13) demonstrate how the charge balance is affected by introducing mono‐, di‐, and trivalent cations as well as resulting vacancies into the β‐eucryptite crystal using Kröger‐Vink notation [33]. As most of the impurities are divalent cations, the substitution mechanism according to Equation (9) is likely the most prominent.

(7) $${\mathrm{Li}}^{+}⇆M^{+}$$

(8) $${\mathrm{Li}}_{Li}^{x}\overset{M^{+}}{↔}M_{Li}^{x}$$

(9) $${\mathrm{Li}}^{+}⇆M^{2+}+V$$

(10) $${\mathrm{Li}}_{Li}^{x}\overset{M^{2+}}{↔}M_{Li}^{•}+V_{Li}^{\prime }$$

(11) $${\mathrm{Li}}^{+}+{\mathrm{Si}}^{4+}⇆\;M^{2+}+N^{3+}$$

(12)

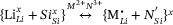




or,

(13)

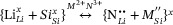




##### 3.1.1.2 Results of the LA‐ICP‐MS Measurements and Lithium Determination

**FIGURE 6:**
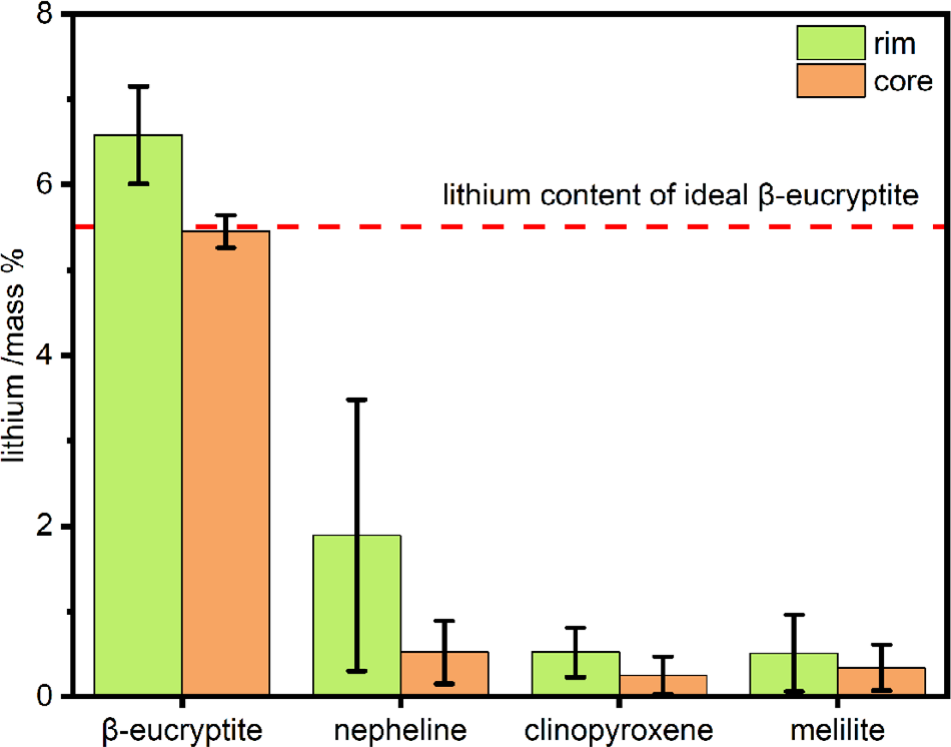
Distribution of lithium in the silicate minerals relative to the sampling position with standard deviation. “Rim” samples represent the outer samples from the slag cone (01b‐01d, 04, 05), while “core” samples represent the central samples (01e, 01f, 02, 03).

Even with the chosen small crater diameter, determining the composition of the β‐eucryptite as well as nepheline by LA‐ICP‐MS proved to be difficult. Consequently, the LA‐ICP‐MS data were clustered in two groups, representing their position in the sampled slag cone. Samples 01b, 01c, 01d, 04, and 05 constitute the group of samples taken from the rim of the slag cone, while samples 01e, 01f, 02, and 03 represent the core. All analyzed minerals show a higher lithium content and a greater standard deviation in the samples from the rim compared to the samples from the core (Figure 6). Consequently, at the rim is a lower proportion of high‐lithium phases, and all phases incorporate more lithium, which demonstrates that the system is further away from an equilibrium state. Hence, the more slowly cooled slag in the center of the slag cone shows a higher enrichment of lithium in the β‐eucryptite compared to the more rapidly cooled samples from the rim of the slag cone.

The β‐eucryptite shows the highest lithium content with an average of 6.58 ± 0.57 mass % in the rim and 5.45 ± 0.19 mass % in the center of the slag cone. Additionally, the β‐eucryptite from the center of the slag cone is very close to the ideal stoichiometric lithium content (5.51 mass %), indicating a near stoichiometric composition with insignificant impurities on the lithium position.

The average composition (Table 1) of the β‐eucryptite was used for determining the lithium content of β‐eucryptite using the mass balance of the EPMA data as there was no significant difference between the main components in the core and rim populations of β‐eucryptite detected. The resulting lithium content is 3.36 mass %, deviating nearly 40% from the direct measurement by LA‐ICP‐MS. However, only convex β‐eucryptite grains with a diameter > 24 µm could be measured with LA‐ICP‐MS, while EPMA measurements were taken of grains with a diameter > 5 µm. Hence, some of the differences between the two results could also reflect a variability in composition depending on grain size.

The β‐eucryptite composition, as determined from the EPMA data and normalized to 4 oxygen anions, is □_0.48_Li_0.46_Al_0.76_Si_1.31_O_4_, where □ denotes a crystallographic vacancy, which is charge balanced. However, adding the average lithium value measured in the β‐eucryptite from core samples, the resulting charge balanced formula would be □_0.29_Li_0.72_Al_0.73_Si_1.26_O_4_. The formula based on the mass balance needs complex substitutions, while the second composition follows a substitution according to Equation (1) which is found in literature as a common substitution in β‐eucryptite, supporting the results of the LA‐ICP‐MS lithium measurements [12].

#### 3.1.2 Composition of the Non‐Lithium Slag Phases

The slag shows a complex mineralogical composition similar to other non‐ferrous slags [34] and its composition is in good accordance with the results of the qualitative bulk x‐ray diffraction (XRD) measurements, published previously [10]. The chemical compositions derived from the EPMA analysis are shown in Table 2, the lithium content of the silicates is displayed in Figure 6.

**TABLE 2:** Mean composition of the main non‐lithium‐containing slag phases measured by EPMA (mass %) with standard deviation in brackets and number of measured grains (n).

				Zoned Fe,Cr,V‐rich spinel group minerals				
Mineral	Nepheline	Clinopyroxene	Melilite	Chromite	Chromite‐Coulsonite ss	Coulsonite	Spinel	Amorphous phase
n	65	104	96	25	47	14	2	10
SiO_2_	46.36 (2.66)	39.29 (1.38)	41.43 (0.8)	0.1 (0.1)	0.22 (0.29)	1.12 (1.19)	0.1 (0.08)	40.3 (0.25)
TiO_2_	0.02 (0.03)	1.42 (0.39)	0.02 (0.02)	0.2 (0.16)	0.38 (0.28)	3.32 (1.2)	0.07 (0.01)	0.11 (0.04)
Al_2_O_3_	34.56 (1.37)	16.23 (1.23)	13.88 (1.17)	11.2 (5.58)	13.41 (2.28)	8.21 (5.19)	51.04 (0.88)	16.45 (0.15)
Cr_2_O_3_	0.02 (0.02)	0.38 (0.1)	0.02 (0.02)	49.85 (5.37)	33.41 (7.03)	3.57 (2.82)	22.45 (5.23)	0.1 (0.03)
V_2_O_3_	0.07 (0.13)	4.8 (1.22)	0.11 (0.05)	6.68 (4.22)	20.41 (3.63)	26.46 (5.0)	1.23 (0.48)	1.19 (0.09)
FeO	0.62 (0.24)	5.18 (1.4)	2.14 (0.53)	14.57 (3.35)	15.69 (2.94)	44.27 (9.4)	8.96 (0.93)	3.67 (0.11)
MnO	0.09 (0.15)	0.63 (0.28)	0.44 (0.2)	2.96 (0.6)	3.25 (0.62)	6.45 (1.19)	1.82 (0.05)	1.5 (0.04)
MgO	0.07 (0.14)	7.69 (0.73)	3.75 (0.9)	7.23 (1.39)	7.2 (0.86)	2.66 (1.52)	11.0 (2.21)	1.03 (0.15)
CaO	2.39 (0.88)	24.25 (0.55)	32.39 (1.29)	0.43 (0.17)	0.53 (0.23)	0.8 (0.58)	0.1 (0.08)	20.51 (0.1)
ZnO	0.08 (0.05)	0.14 (0.09)	1.02 (0.52)	6.15 (0.91)	6.38 (0.62)	2.48 (1.36)	3.9 (1.38)	< LOD
BaO	0.13 (0.15)	0.02 (0.02)	0.03 (0.03)	0.03 (0.04)	0.05 (0.06)	0.19 (1.52)	0.03 (0.03)	0.84 (0.04)
Na_2_O	15.29 (0.56)	0.33 (0.12)	4.5 (0.58)	0.14 (0.07)	0.17 (0.08)	0.16 (0.19)	0.09 (0.03)	8.55 (0.25)
K_2_O	0.9 (0.21)	0.01 (0.01)	0.02 (0.02)	< LOD	0.01 (0.01)	0.02 (0.01)	< LOD	0.33 (0.01)
Cl	0.01 (0.03)	< LOD	< LOD	< LOD	0.01 (0.01)	< LOD	< LOD	0.33 (0.01)
Total	100.6 (1.14)	100.36 (0.54)	99.76 (0.67)	99.54 (0.83)	101.09 (0.79)	99.71 (1.84)	100.78 (0.75)	94.84 (0.45)

The nepheline shows a sodium deficiency, excess SiO_2_, and additional CaO, resulting in a nepheline with a notable quartz and anorthite component [35]. Furthermore, the nepheline incorporates by far the most potassium of all crystalline slag phases. The lithium content of the nepheline from the rim has an average of 1.9 ± 1.6 mass % lithium but decreases substantially to 0.52 ± 0.37 mass % in the core samples (Figure 6). This is expected, as nepheline can substitute up to 22% of β‐eucryptite into its structure as a solid solution resulting in a nepheline of the composition Li_0.23_Na_0.77_AlSiO_4_ [36].

Based on its composition, the clinopyroxene can be categorized as a vanadian aluminian subsilicic augite [37]. However, Whitley et al. described a naturally occurring, very similar clinopyroxene as calcium tschermakite, albeit with significantly lower vanadium content [38]. For the sake of simplicity, this mineral will nonetheless be referred to as clinopyroxene. The melilite can be described as a gehlenite containing components of åkermanite, soda‐melilite, and iron‐melilite [39]. The clinopyroxene and melilite contain the lowest amount of lithium with around 0.5 mass % in the rim and 0.3 mass % in the core, respectively.

**FIGURE 7:**
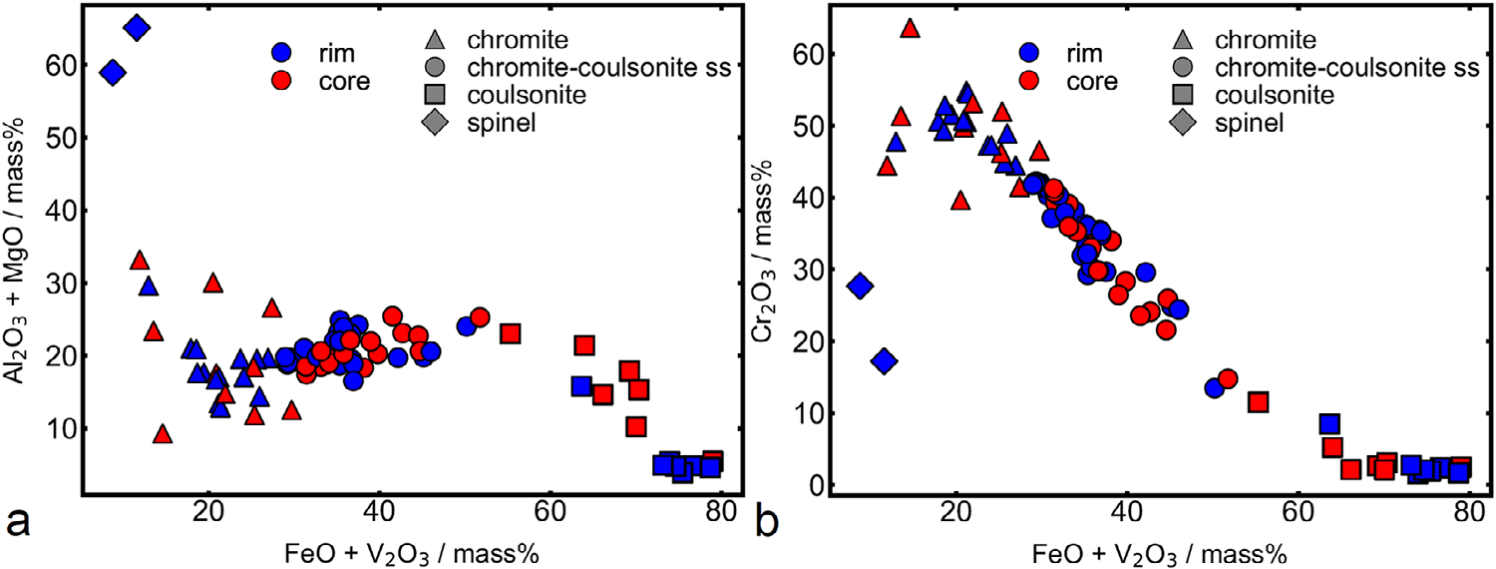
Visualization of the different mineral chemistries of the detected spinel group minerals with respect to their occurrence in the slag cone: Chromite, chromite‐coulsonite solid solution, coulsonite, and spinel. (a): FeO + V_2_O_3_ plotted against Al_2_O_3_ + MgO. (b): FeO + V_2_O_3_ plotted against Cr_2_O_3_.

**FIGURE 8:**
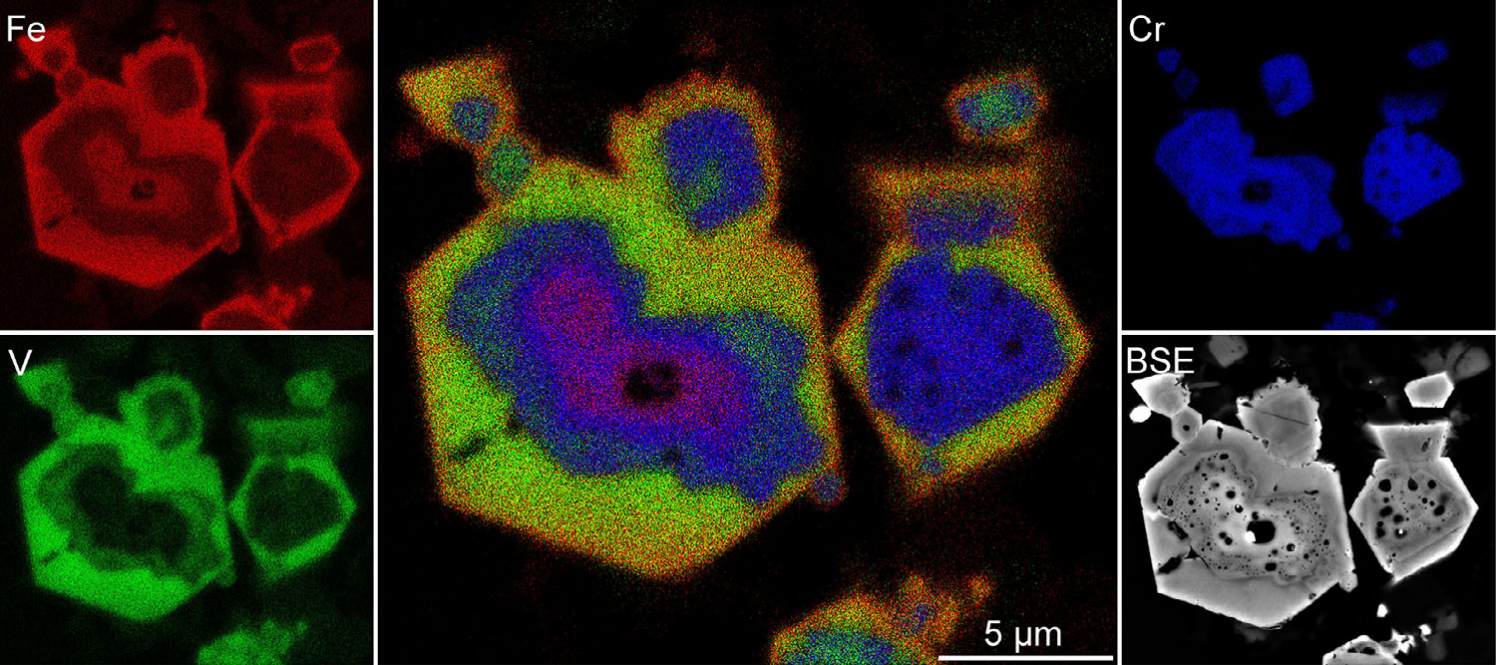
EDX mapping of a zoned Fe, Cr, V‐rich spinel group mineral from sample 02. The inner zonation shows chromite composition, the outer zonation shows coulsonite composition, and the intermediate zonation shows a mixed composition with V alongside Cr.

The slag shows two distinct spinel group minerals in different quantities. Based on their main components they can be classified as the chromite (FeCr_2_O_4_) – coulsonite (FeV_2_O_4_) solid solution series (Figure 7) and spinel (MgAl_2_O_4_) [40]. Only two specimens of MgAl_2_O_4_‐spinel could be detected, making it a rare accessory phase in the slag that still contains high amounts of Cr_2_O_3_. Chromite – coulsonite solid solutions are by far the most abundant spinel group mineral in the slag. Using the “Uniform Manifold Approximation and Projection” (UMAP) approach in the software ioGAS ver. 8.2 [41], it was possible to cluster the phases from the chromite – coulsonite solid solution into 3 groups: chromite, coulsonite, and solid solution of intermediate composition. These three groups are frequently found together as zonation of the same grain with an inner core of chromite, an outer rim of coulsonite and the intermediate composition in between (Figure 8). Further, it should be noted that there is no dependence between the mineral chemistry of the spinel and the occurrence in the slag cone (Figure 7). This specific layered spinel is already described for similar slags [42].

Chromium is almost exclusively found in the spinel group minerals, with minor amounts in the clinopyroxene (0.38 ± 0.1 mass %). Similar is true for vanadium, albeit with a notable content of V_2_O_3_ in the clinopyroxene (4.8 ± 1.22 mass %). Thus, chromium and vanadium are of no concern for the lithium extraction based on a β‐eucryptite concentrate.

### 3.2 Microstructure

**FIGURE 9:**
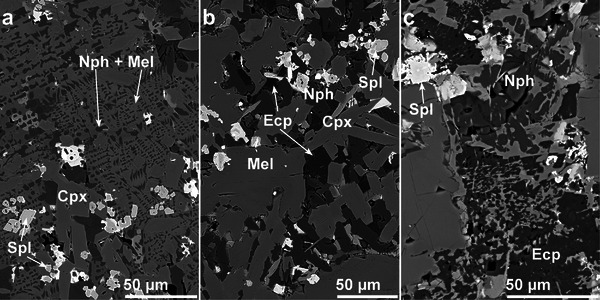
SEM‐BSE images of different microstructures. (a): Euhedral spinel and clinopyroxene crystals surrounded by graphically intergrown melilite and nepheline (sample 01c). (b): Rectangular melilite and euhedral clinopyroxene crystals host subhedral nepheline and some subhedral β‐eucryptite as interstitial phases (sample 01d). (c): Nepheline and β‐eucryptite show anhedral crystals due to strong intergrowth with the amorphous phase (sample 02).

Based on the microstructure and mineral composition of the different samples, the crystallization sequence of the slag can be unraveled. The euhedral crystal shapes of the spinel and clinopyroxene in samples 01c, 04, and 05 indicate that these phases form earliest. Often, clinopyroxene crystals can be observed around a spinel crystal, suggesting that the clinopyroxene used the spinel as a substrate for heterogeneous nucleation, a phenomenon that is supported by literature [43]. In these samples, nepheline and melilite exhibit a graphic intergrowth (Figure 9a). In sample 01d, a different microstructure can be observed. The melilite forms rectangular crystals enclosing the earlier precipitated spinel and clinopyroxene. The nepheline and the rarely occurring β‐eucryptite form mostly convex subhedral crystals as interstitial phases (Figure 9b). The β‐eucryptite can be more frequently observed in samples taken further away from the rim of the slag cone, indicating a dissolution of β‐eucryptite from nepheline if cooling rates are sufficiently low (Figure 9c). This is supported by the LA‐ICP‐MS data, as it yielded higher lithium levels in the nepheline from the “rim” specimens compared to the “core” specimens. In the following paragraphs, the grain shape and grain size distribution of β‐eucryptite and the spinel phases will be quantitatively analyzed.

#### 3.2.1 Volume Fraction and Grain Shape Analysis

Ideal for the liberation of a grain is a fully convex shape with little elongation, usually best achieved by an euhedral crystal. This translates to an aspect ratio close to 1, combined with a low excess perimeter (*deltP*) and excess area (*deltA*) in terms of shape descriptors. To obtain more nuanced information, the data were split into two subgroups: grains above and below a grain size of 10 µm eq. diameter, as this is a reasonable lower size limit of flotation, a common method for mineral separation that has already shown promise for the separation of β‐eucryptite from melilite [18, 44]. The measured area % of any phase in the 2D section is equivalent to the volume % of that phase in the 3D sample volume, because the samples were cut in an arbitrary orientation [45]. The final volume fractions were derived by normalizing the phase maps of β‐eucryptite and spinel with the pore phase maps (Table 3).

**TABLE 3:** Volume fraction with error (Δϕ_2_) and shape descriptors (aspect ratio, *deltP*, *deltA*) of β‐eucryptite and spinel greater and smaller than 10 µm eq. diameter.

sample			01c	01d	01e	01f	02	03	Mean
spinel	volume fraction [vol %]		4.73 ± 0.13	5.32 ± 0.14	5.60 ± 0.15	5.60 ± 0.19	6.40 ± 0.40	5.61 ± 0.19	5.52 ± 0.20
	mean aspect ratio	> 10 µm	1.88	1.88	1.86	1.86	1.87	1.86	1.87
		< 10 µm	1.95	2.03	2.00	2.06	2.08	1.97	2.02
	mean *deltP* [%]	> 10 µm	24.3	20.1	18.4	18.9	22.6	19.2	20.6
		< 10 µm	2.7	1.9	2.0	2.3	2.1	2.6	2.3
		global	4.5	3.8	4.7	4.3	4.8	5.2	4.5
	mean *deltA* [%]	> 10 µm	51.1	43.8	38.1	38.3	39.9	37.1	41.4
		< 10 µm	34.7	34.0	29.9	32.6	32.0	32.6	32.6
		global	36.0	35.0	31.2	33.3	33.1	33.3	33.6
β‐eucryptite	volume fraction [vol %]		—	2.26 ± 0.10	2.45 ± 0.17	5.28 ± 0.25	6.68 ± 0.19	4.85 ± 0.32	4.31 ± 0.21
	mean aspect ratio	> 10 µm	—	2.21	2.15	2.13	2.03	2.12	2.13
		< 10 µm	—	2.51	2.13	2.19	2.19	2.34	2.27
	mean *deltP* [%]	> 10 µm	—	19.5	20.8	30.3	36.2	24.5	26.2
		< 10 µm	—	0.9	1.3	2.3	2.7	1.4	1.7
		global	—	1.6	2.4	4.6	5.6	3.2	3.5
	mean *deltA* [%]	> 10 µm	—	60.4	53.0	47.3	64.0	51.9	55.3
		< 10 µm	—	46.7	37.5	42.7	43.2	42.3	42.5
		global	—	47.2	38.3	43.9	45.0	43.0	43.5

For both phases, an uneven distribution can be observed. The average β‐eucryptite content in the slag cone is 4.31 ± 0.21 vol %. However, it can be observed that the β‐eucryptite content increases from the rim to the center of the slag cone, with no notable difference between a sampling depth of 20 cm (sample 01f) and the center (sample 03). The highest amount of β‐eucryptite can be found in the tip of the slag cone (6.68 ± 0.19 vol %). The spinel content is similar for most of the slag cone, displaying quantities close to the average of 5.52 ± 0.20 vol %. Only the most outer sample (sample 01c) and the tip of the slag cone (sample 02) contain deviating values with a spinel content of 4.73 ± 0.13 vol % and 6.40 ± 0.40 vol %, respectively.

The aspect ratios of β‐eucryptite and spinel grains are both slightly increased in the < 10 µm fraction compared to the > 10 µm fraction. However, the spinel shows much less variation in the aspect ratios of the respective size fractions and lower aspect ratios in general compared to the β‐eucryptite.

**FIGURE 10:**
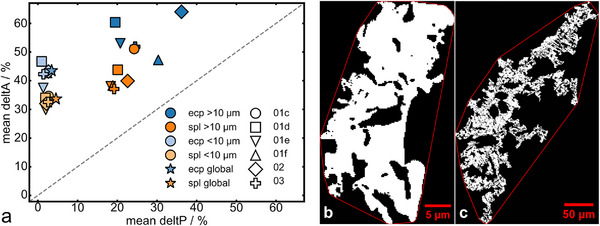
(a) Plot of the mean *deltP* (%) : mean *deltA* (%) of the grain size fraction > 10 µm and < 10 µm eq. diameter of the β‐eucryptite and spinel of the individual samples. b: Exemplary image of the envelope (red line) of a small β‐eucryptite grain with low *deltP* and high *deltA* and c: of a large β‐eucryptite grain with high *deltP* and *deltA*.

The envelope parameters of β‐eucryptite and spinel show significant differences between the two size fractions (Figure 10). While the < 10 µm fraction of both phases shows a similarly low *deltP* with 0.9% – 2.7% for β‐eucryptite and 1.9% – 2.7% for spinel, the > 10 µm fractions exhibit different trends. The spinel displays similar, moderate *deltP* values across all samples (18.4% – 24.3%). However, β‐eucryptite shows a drastic increase in *deltP* with sampling position, resulting in almost double the excess perimeter in sample 02 compared to 01d (19.5% to 36.2%). Conversely, sample 03 shows a lower *deltP* than sample 01f and 02, hinting at a reduction in excess perimeter towards the center of the slag cone. The average global *deltA* of β‐eucryptite (43.6%) is significantly higher compared to spinel (33.7%). Interestingly, the *deltA* of spinel decreases from sample 01c to 03, from 51.1% to 37.1% in the > 10 µm fraction, where it then remains relatively constant. The same trend is not present in the < 10 µm fraction. The β‐eucryptite shows high variation in *deltA* of up to 16.7% in the > 10 µm fraction; however, no consistent trend can be detected.

The shape descriptors show that the spinel is, on average, slightly elongated, with a low global excess perimeter and a moderately high excess in area; the latter can likely be attributed to the internal rounded cavities common among the spinels (Figures 8, 9). These features can drastically increase *deltA* with minimal influence on *deltP*. Hence, the decrease in *deltA*, unique to the spinel, is likely a result of a reduction in internal holes with increasing sampling position. The β‐eucryptite grains exhibit a slightly more elongated shape. They also display a similarly low global *deltP* but a high *deltA*, about 10% greater than the spinel. The high *deltA* of the β‐eucryptite is likely a result of internal cavities as well as lobate grain boundaries (Figures 9, 10). In general, sample 03 resembles sample 01f in grain shape and volume fraction, implying little change in the slag between 20 cm and the center of the slag cone. However, a notable difference in volume fraction and grain shape of both investigated phases for samples 02 and 03 indicates different cooling mechanisms in the tip of the slag cone (sample 02) and the most inner core (sample 03), with sample 02 displaying, on average, more β‐eucryptite and spinel with higher *deltP* and *deltA* compared to sample 03. Consequently, cooling of a slag cone produces top‐down zonation in addition to an internal‐external zonation.

All measured samples of both grain size fractions show a higher *deltA* than *deltP*, which is favorable for recovery, as crack‐like or dendritic structures, strongly increasing the *deltP* with minimal increase in *deltA*, are more difficult to process than grains with concave areas. Nevertheless, the β‐eucryptite grains > 10 µm show such a high *deltA* that efficient liberation with conventional methods is likely still very difficult. In contrast, the spinel grains < 10 µm display a more favorable shape with a lower *deltA*.

#### 3.2.2 Grain Size Distribution

**FIGURE 11:**
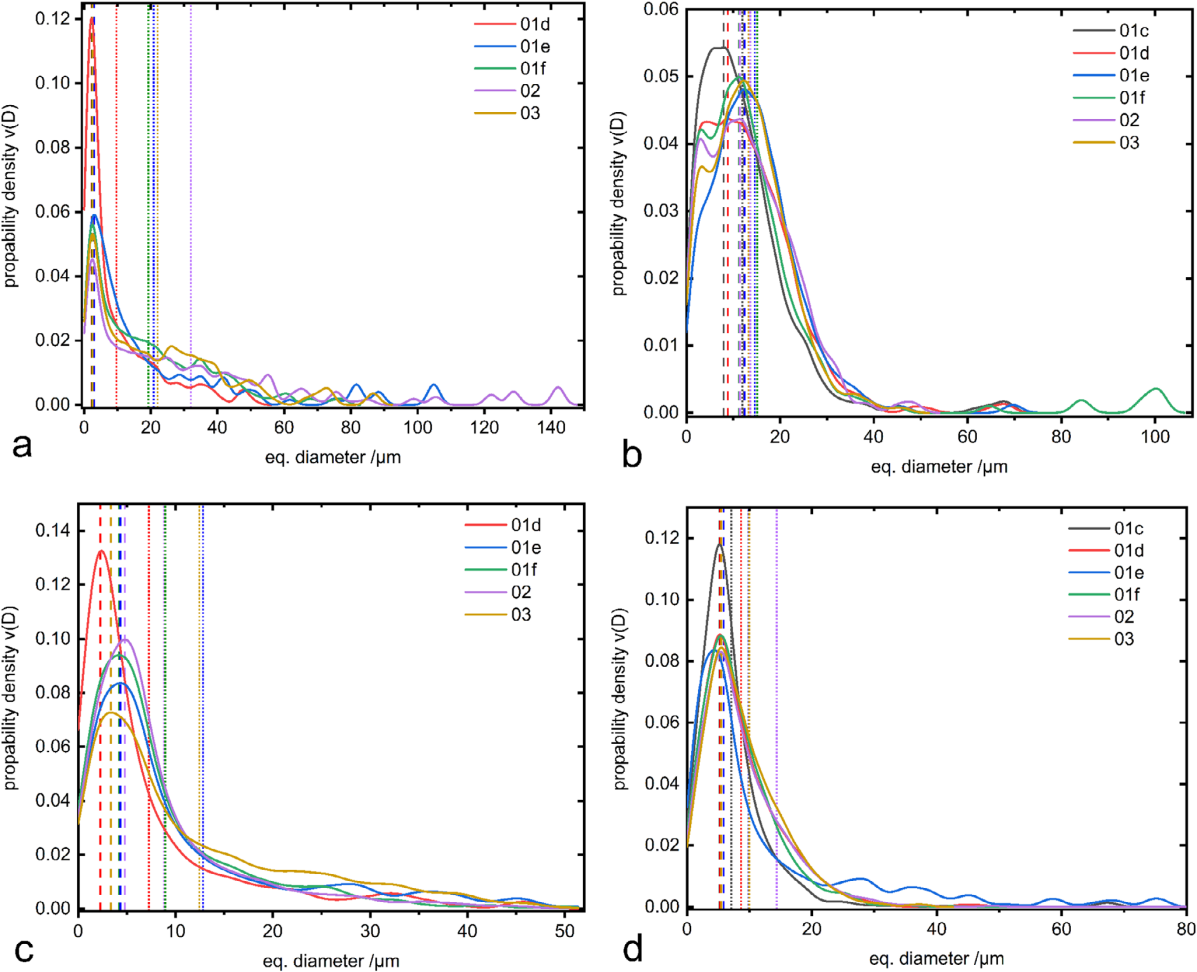
3D grain size distribution of β‐eucryptite (a) and spinel (b) based on the original phase maps. 3D grain size distribution of β‐eucryptite (c) and spinel (d) based on the phase maps after applying a watershed. The dashed lines represent the mode of the grain size, the dotted lines the mean grain size.

Based on the phase maps, a 3D grain size distribution of β‐eucryptite and spinel (Figure 11a,b) was calculated from the area sections of these phases [45]. Additionally, the 3D grain size distribution was calculated again after applying a watershed algorithm on the phase maps in order to separate mineral clusters and simulate more realistic grain sizes after comminution of the slag (Figure 11c,d). An overview of all relevant calculated grain size measures is given in Table 4.

**TABLE 4:** The volume fraction *f_min_* > 10 µm and different grain size measures for β‐eucryptite and spinel in the samples and after applying a watershed algorithm.

Sample		01c	01d	01e	01f	02	03	Mean
spinel	volume fraction *f_min_* > 10 µm [%]	50.0	58.3	61.3	55.9	61.1	60.7	57.9
	watershed: volume fraction *f_min_* > 10 µm [%]	15.5	30.4	35.0	26.7	31.9	30.9	28.4
	area‐weighted mode *f_min_* [µm]	7.3	6.3	8.6	9.6	9.4	8.2	8.2
	watershed: area‐weighted mode *f_min_* [µm]	3.6	4.3	4.3	4.2	3.8	4.4	4.1
	mean grain size v(D) eq. diameter [µm]	12.0	13.6	14.6	15.1	13.7	13.3	13.8
	watershed: mean grain size v(D) eq. diameter [µm]	7.1	8.7	9.9	10.0	14.4	8.9	10.0
β‐eucryptite	volume fraction *f_min_* > 10 µm [%]	—	49.7	57.0	70.8	78.5	74.0	64.0
	watershed: volume fraction *f_min_* > 10 µm [%]	—	35.5	42.1	31.3	28.4	50.1	34.3
	area‐weighted mode of *f_min_* [µm]	—	1.1	3.5	3.3	5.0	2.3	3.2
	watershed: area‐weighted mode of *f_min_* [µm]	—	2.0	3.2	3.3	3.1	3.6	3.0
	mean grain size v(D) eq. diameter [µm]	—	9.7	20.9	19.3	32.1	22.0	20.5
	watershed: mean grain size v(D) eq. diameter [µm]	—	7.3	12.8	9.0	8.8	12.4	9.5

The volume fraction *f_min_* > 10 µm of both phases increases with increasing depth of the sampling position, demonstrating a general trend. However, for both samples, these values are significantly reduced after applying a watershed. Interestingly, the β‐eucryptite shows a trend of decreasing volume fraction *f_min_* > 10 µm with increasing sampling depth after the watershed algorithm is applied. The exception is sample 03 displaying the highest value of volume fraction *f_min_* > 10 µm of any phase or sample. For the spinel, the decrease in volume fraction *f_min_* > 10 µm after application of the watershed algorithm is most drastic for sample 01c, where the volume fraction is reduced by a factor of 3.2, while the other samples’ volume fraction is only nearly halved. This fits with the high *deltA* of sample 01c produced by an increased amount of internal cavities. The watershed algorithm exploits these holes in the spinel grains to break up individual particles, which otherwise would not be altered by the watershed algorithm.

The mean grain size was also included as a measure because it is probably the most prominent grain size measure in literature. It is plotted alongside the mode in Figure 11, which distinctly illustrates the overall reduction in grain size by breaking up mineral clusters and larger lobate grains.

The area‐weighted mode is a reliable measure of which grain size class contributes the most area to the overall area of the phase. For the β‐eucryptite, the area‐weighted mode of the original does not change drastically compared to the watershed‐segmented version. However, the watershed area‐weighted mode grain size shows far less variation, displaying a constant grain size for samples 01e – 03 more clearly than the original.

For the spinel, the area‐weighted mode is constantly about halved by the watershed algorithm from 7.3 – 9.6 µm to 3.6 – 4.4 µm. This shows that an easy‐to‐interpret increase in the area‐weighted mode of the original phase map is merely a tendency of the spinel to form mineral clusters that are deconstructed by the watershed algorithm, revealing a mostly constant area‐weighted mode of the grain size. Additionally, the mean grain size as well as the watershed mean grain size show strong variation while with no coherent trend can be discovered also indicating a constant mean grain size. This demonstrates the sensitivity of the mean grain size as a grain size measure in comparison to the more robust area‐weighted mode, as the mean is way more prone to being distorted by outliers or extreme values.

The β‐eucryptite displays an increasing difference between the two mean grain sizes with increasing sampling depth, with the highest measured difference in the tip of the slag cone (sample 02). An overall increase in grain size and volume fraction *f_min_* > 10 µm with increasing sampling depth is observed. However, the β‐eucryptite shows a drastic decrease of all grain size measures when exposed to a watershed algorithm. This aligns well with the highly lobate grain shapes quantified previously. Both phases show a very low overall grain size after the application of the watershed algorithm with about 34.3% (β‐eucryptite) and 28.4% (spinel) of the volume fraction being < 10 µm and thus of sufficient size for a potential subsequent froth flotation.

### 3.3 Effects of Microstructure and Crystal Chemistry on the Recyclability of the β‐Eucryptite and Spinel

The EnAM strategy relies on enriching a target element like lithium in a specific phase to increase its content in the recycling material by producing a concentrate. The 5.45 mass‐% lithium, which is the maximum lithium concentration possible in a pure β‐eucryptite concentrate, would increase the average lithium content of a primary spodumene concentrate when mixed. Furthermore, most potentially problematic cations are effectively removed alongside the minerals they are incorporated in (e.g., Cr in the spinel group minerals). In a co‐processing of a secondary β‐eucryptite concentrate with a primary spodumene concentrate, the hydrometallurgical impact of cationic impurities of β‐eucryptite can be evaluated based on the primary lithium refining process of spodumene.

Iron and calcium are the only impurities of relevant quantity that the pyrometallurgical β‐eucryptite introduces to the process. As both elements are also typical impurities in spodumene, the refining process already features mechanisms to remove those impurities, for example, by filtering the solution [14]. However, iron tends to capture lithium, subsequently reducing lithium yields. Thus, various processes to remove iron are explored [14].

To produce a β‐eucryptite concentrate, e.g. by flotation, an efficient liberation of the grains must be achievable [18]. The microstructure of the investigated β‐eucryptite is characterized by comparatively small grain sizes, that are further reduced when a realistic grain shape after comminution is considered. The resulting grain sizes are mostly below the lower size threshold of flotation. Furthermore, the high *deltA* indicates high amounts of intergrowth with non‐lithium‐containing phases. These factors make efficient liberation with conventional crushing methods difficult.

Accumulating the spinel into a concentrate, for example by implementing a second flotation step, and thus separating it from the residual silicate phases would have two beneficial effects [46]. First, a spinel concentrate could be the basis for recovering the chromium and vanadium present in the slag [42, 47]. Second, the removal of the spinel and thus the removal of chromium and vanadium from the slag increases the quality of the by‐products. However, the spinel shows a similarly low grain size, which render most of the spinel too fine for subsequent flotation.

The by‐product, produced by separating a β‐eucryptite‐ and a spinel concentrate, consist of the non‐lithium‐containing silicates melilite, clinopyroxene, and nepheline. The chemical composition of the by‐product is rich in SiO_2_, CaO, and Al_2_O_3_ and, due to the removal of the spinel fraction, shows a reduced heavy metal content. This improved chemical composition makes it potentially attractive for the cement industry as either filler or supplementary cementitious material, depending on its reactivity [48, 49].

## 4 Conclusions

The investigated slag contains β‐eucryptite, a potential lithium accumulating EnAM‐phase. The investigated β‐eucryptite contains only minor amounts of impurities, mostly CaO and FeO, which are already handled in the lithium refining process from primary spodumene. The lithium content in the β‐eucryptite and the non‐lithium‐containing silicates is higher in the rim of the slag cone compared to the center, which would result in a high lithium loss. The β‐eucryptite from the core of the slag cone has nearly the stoichiometric lithium content and there is considerably less lithium in the nepheline and other non‐lithium‐containing silicates. Hence, a higher recycling efficiency could be achieved with the material from the core of the slag cone.

The β‐eucryptite content increased gradually toward the center of the slag cone, with the highest measured content of 6.68 ± 0.19 vol % in sample 02. However, with increasing sampling depth, the excess perimeter (*deltP*) of the β‐eucryptite also increased, most notably for larger grains from 19.5% to 36.2%. Together with a constantly high excess area (*deltA*), averaging around 43.6% the grain shape is unfavorable for separation and subsequent recovery. The influence of the complex grain shape was considered by comparing the grain sizes of the original phase map to a phase map that was altered using a watershed algorithm to disassemble highly lobate grains and mineral clusters, which would be broken up during comminution. While the area‐weighted mode of the grains did not significantly change, the calculated 3D mean grain size was reduced substantially by about 50%, resulting in a considerable reduction in β‐eucryptite, sufficiently large for flotation, to around 34.3%.

**FIGURE 12:**
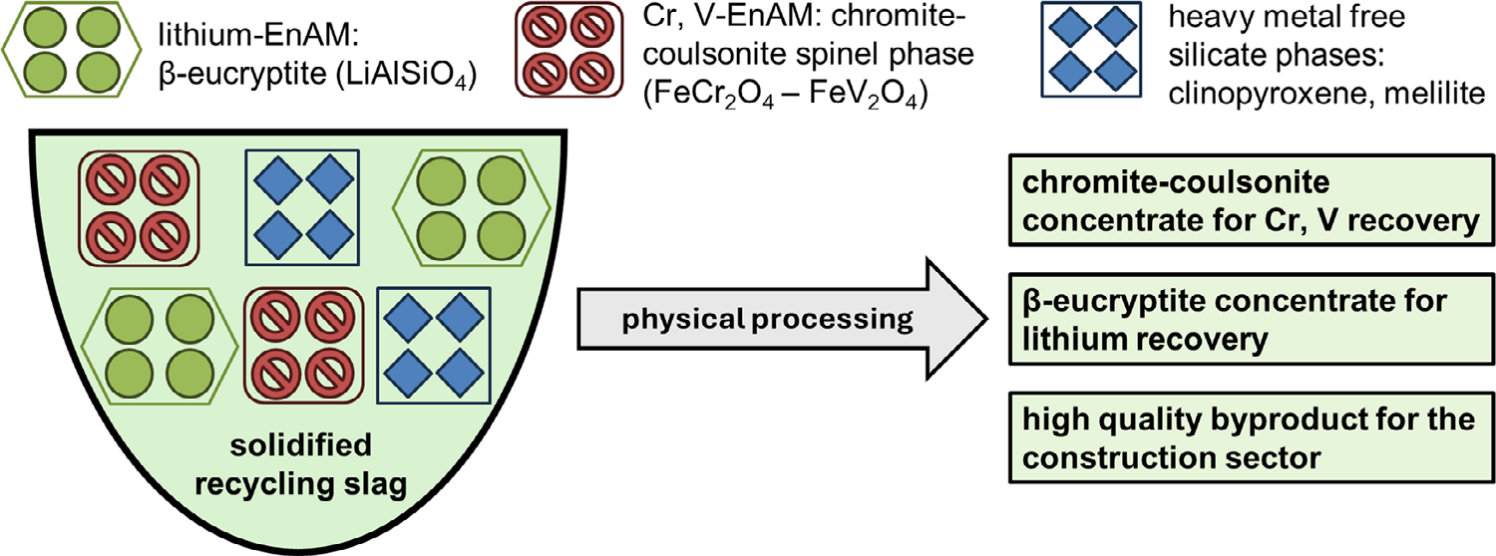
Circular approach for the valorization of all phases contained in the battery recycling slag.

The spinel was identified as containing most of the chromium and vanadium in the slag. Hence, removing the spinel from the residual slag phases, one could potentially recover chromium and vanadium and reduce their respective contents in the residual slag, enabling the upcycling of the residual material (Figure 12). However, while the excess perimeter averages a low 4.4%, the excess area averaged around 33.7%, which is lower compared to the β‐eucryptite but remains high. The grain shape led to a reduction of spinel of floatable size from 57.3% to 27.9%.

To achieve a slag with more favorable properties for lithium recovery, slag conditioning should be employed. In general, the goal should be to achieve a slag with larger grain sizes and more convex β‐eucryptite grains and reduce cavities in the spinel grains. Fruitful approaches could involve modifying the slag composition or optimizing the cooling conditions. Practical examples for these approaches are given by Mou et al. 2019, who showed how increasing the Fe_2_O_3_ content in a steel slag increases the size of the present spinels and by Tsunazawa et al. 2019, demonstrating that decreased cooling rates increase weight fraction and grain size of magnetite in copper smelting slags [50, 51]. Furthermore, simplifying the slag composition by reducing the total number of phases could also prove beneficial. Additionally, advanced comminution methods or specialized flotation could further enable holistic recycling. Furthermore, this study emphasizes the importance of considering microstructure when investigating the recycling potential of a pyrometallurgical slag. It is demonstrated that a promising mineralogical composition can still be difficult to process, if grain sizes are small or grain shapes are too complex.

## Author Contributions

**Peter Cornelius Gantz**: Writing – original draft (lead); data curation (lead); investigation (lead); formal analysis (lead); methodology (lead); visualization (lead); **Charlize Alexia Senkyr**: Visualization (supporting); data curation (supporting); Writing – review and editing (supporting); **Rüdiger Kilian**: Methodology (supporting); Writing – review and editing (equal); **Andreas Neumann**: Writing – review and editing (equal); **Maximilian Korges**: Methodology (equal), Writing – review and editing (supporting); **Daniel A. Frick**: Methodology (supporting), data curation (supporting), Writing – review and editing (equal); **Hans Roggendorf**: Supervision (equal); Writing – review and editing (equal); **Ralf Wehrspohn**: Conceptualization (lead); visualization (supporting); supervision (equal); Writing – review and editing (equal); **Stefan Stöber**: Project administration (lead); supervision (equal); Writing – review and editing (supporting); **Christiane Stephan‐Scherb**: Visualization (supporting); supervision (equal); Writing – review and editing (lead).

## Ethical Statement

This research was conducted in full compliance with ethical standards and principles. No human participants or human tissue samples were involved in any part of this research. Additionally, no animals were used or harmed during this study.

## Conflicts of Interest

The authors declare no conflicts of interest.

## Supporting information




**Supporting File**: advs74329‐sup‐0001‐SuppMat.docx.

## Data Availability

The data that support the findings of this study are available from the corresponding author upon reasonable request.
