# Supplementary material for: Circular Potential of Lithium‐Ion Battery Recycling Slags: Quantifying Microstructure and Elemental Distribution for a Holistic Valorization
Source: Adv Sci (Weinh). 2026 Feb 11;13(22):e23988. doi: 10.1002/advs.202523988 (PMC13088323; doi:10.1002/advs.202523988)
Supplement: Supplementary file 1 — Supporting File: advs74329‐sup‐0001‐SuppMat.docx. [file ADVS-13-e23988-s001.docx]

Supporting Information

Lithium Distribution and Microstructural Analysis of Lithium-Ion Battery Recycling Slags using In-Situ Methods

Peter Cornelius Gantz*, Charlize Alexia Senkyr, Rüdiger Kilian, Andreas Neumann, Michael Neumann, Maximilian Korges, Daniel A. Frick, Hans Roggendorf, Ralf Wehrspohn, Stefan Stöber, and Christiane Stephan-Scherb*

Table S1. Step-by-Step documentation of the performed image analysis.

| **Analysis** | **Steps** | **Description** |
| --- | --- | --- |
| **Pre-Processing** | Mean shift | Applied a mean shift as edge preserving filter. |
|  | Brightness Contrast | Applied B/C to set the gray value of the pores to 0. |
|  | Pen | Removed polish residue from pores if necessary. |
| **Segmentation** | Grey value thresholding | Threshold to separate β-eucryptite, spinel, and pores, respectively into a binary phase map. |
| **Post-Processing** | Remove outliers (bright) | Standard settings (2/50) |
|  | Analyze particles | Set size threshold to 1-infinity [µm²] removing particles smaller 26 px 🡪 splitting the phase map into two size fractions 🡪 result was only used for shape analysis (aspect ratio + deltP/deltA). |
| **Measurement** | Analyze particles + Jazy_Error | Measurement of area fraction 🡪 determination of the error. |
| **Grain shape analysis** | measurement of the aspect ratio | Set Measurements 🡪 Shape descriptors, Feret’s diameter; Analyze particles 🡪 show masks 🡪 Summarize, Exclude on edges. |
|  | Jazy_Env_map macro | “map env. prop", applied on phase maps >10 µm eq. diameter and 1<10 µm eq. diameter. |
| **Grain size analysis** | Jazy_stripper macro | Applied the macro with a min. equivalent diameter of 0 µm, a maximum parameter of 150 µm with 15 bins. |
|  | BioVoxxel Toolbox | Applied the plugin with an erosion cycle number of 10. |

Table S2. Overview of the standards used for the calibration of the silicate and oxide phase measurements.

| standard name | mineral name | used to calibrate following elements | source institution |
| --- | --- | --- | --- |
| NMNH 11590 | plagioclase | Al_2_O_3_, Na_2_O, CaO | Smithsonian Institut |
| NMNH 143965 | hornblende | SiO_2_, TiO_2_, Al_2_O_3_, CaO, MgO, FeO, Na_2_O, K_2_O | Smithsonian Institut |
| 19. Chromite | chromite | Cr_2_O_3_ | Astimex Standards Ltd. |
| 9. Vanadium V 99.8% wire | vanadium | V_2_O_3_ | SPI Supplies |
| 50. Tugtupite | tugtupite | Cl | Astimex Standards Ltd. |
| NMNH 86539 | benitoite | BaO | Smithsonian Institut |
| NMNH 145883 | gahnite | MnO, ZnO | Smithsonian Institut |
